# Impact of slab selection on the relationship between choriocapillaris flow deficits and enlargement rate of geographic atrophy

**DOI:** 10.1038/s41433-023-02788-2

**Published:** 2023-10-21

**Authors:** Ahmed Roshdy Alagorie, Giulia Corradetti, Iksoo Byon, Liran Tiosano, Yongsok Ji, Muneeswar Nittala, Swetha Velaga, Marco Nassisi, Aditya Verma, Srinivas R. Sadda

**Affiliations:** 1 Doheny Eye Institute, Pasadena, CA USA; 2 Department of Ophthalmology, David Geffen School of Medicine at UCLA, Los Angeles, CA USA; 3 Department of Ophthalmology, Faculty of Medicine, Tanta University, Tanta, Egypt; 4 Retinal Disorders and Ophthalmic Genetics Division, Stein Eye Institute, David Geffen School of Medicine, University of California, Los Angeles, CA USA; 5 Department of Ophthalmology, Research Institute for Convergence of Biomedical Science and Technology, Pusan National University Yangsan Hospital, Pusan National University School of Medicine, Yangsan, South Korea; 6 Department of Ophthalmology, Chonnam National University Medical School and Hospital, Gwangju, South Korea

**Keywords:** Outcomes research, Geriatrics

## Abstract

**Objective:**

To evaluate the effect of changing slab position on the correlation between choriocapillaris (CC) flow deficits (FD) in eyes with geographic atrophy (GA) and yearly enlargement rate (yER) of GA.

**Methods:**

OCT and OCTA images obtained on Cirrus HD-OCT device were collected from patients with GA. Each patient underwent OCTA scan at baseline and two OCT scans, one at baseline and one after at least 12 months. GA was delineated on en-face fundus image to calculate yER. OCTA images were generated from three 10 µm thick slabs 11, 21 and 31 µm posterior to RPE-fit line. 100 µm-wide concentric rings were generated around GA to calculate FD% in each ring which was correlated with yER.

**Results:**

For the 11–21 µm slab, FD% was not significantly correlated with yER for any of the rings (*p* > 0.05). For the 21–31 and 31–41 µm slab, FD% of rings located in the 600 µm region around GA was significantly correlated with yER (*p* < 0.05). However, in all slab locations, there was no significant correlation between yER and CC FD% of rings located beyond the 600 µm region (*p* > 0.05).

**Conclusions:**

Slab selection for quantification of CC FD% may have a significant impact on quantitative results in eyes with GA.

## Introduction

Choriocapillaris (CC), the innermost part of the choroid, lies posterior to the Bruch’s membrane (BM) and plays a vital role in supporting retinal function. It represents the capillary circulation of the choroid that is responsible for nourishing the outer retinal layers and transporting waste products from the retinal pigment epithelium (RPE) [1]. Although the fine structure of the CC cannot be evaluated by traditional imaging techniques such as optical coherence tomography (OCT), fluorescein angiography or indocyanine green angiography, OCT angiography (OCTA) has allowed the choriocapillaris to be discerned [2].

Quantification of the CC has been a topic of considerable recent interest as CC flow deficits (FD) have been linked to multiple macular disorders including age-related macular degeneration, central serous chorioretinopathy, retinitis pigmentosa and pachychoroid spectrum disease [3–9]. However, assessment and quantification of the CC can be challenging and may be impacted by a variety of factors including signal quality, motion artifacts, and segmentation errors [10]. An additional factor that may affect CC quantitative assessment is the axial location of the slab used to analyse the CC.

At present, there is not yet a consensus for the optimal location and depth of CC slab that should be used for quantitative assessment and different slabs have been used by various research groups [4, 6, 9, 11–13]. As a result, it is difficult to compare the CC FD values from these various studies. Systematic investigations of the impact of the permutations of slab thickness and slab position on CC FD measurements in normal eyes demonstrated that shifts as small as 4 µm, could lead to considerable differences in FD% measurements [14]. This study also demonstrated that slabs close to Bruch’s membrane were more likely to be confounded by artifact, such as regions of hypointensity, due to inadvertent inclusion of the RPE layer as a result of subtle segmentation artifacts [14]. Deeper slabs were more repeatable and less likely to be impacted by these subtle segmentation errors, but deeper slabs are assumed to rely on capturing the projection artifact emanating from the CC, and may be confounded by signal arising from structures present at that deeper slab location. For that reason, some have suggested that the term inner choroidal slab be used to more accurately describe this slab extracted from a physical location deeper than the known histologic position of the CC. Regardless of the descriptive term used, perhaps the more important question is whether the deeper slab can be informative and useful as a clinical biomarker.

CC degeneration has been observed in regions surrounding geographic atrophy (GA) lesions by histopathological studies, and greater CC flow deficit has also been identified adjacent to GA lesions by OCTA [9, 15]. The severity of CC flow impairment in eyes with GA has also been reported to correlate with the enlargement rate of the GA lesion. There is controversy, however, as to which region of the macula is most predictive of the rate of GA enlargement. Whereas, Thulliez and colleagues [16] observed that CC FD in regions remote from the GA lesion were most predictive, we observed that a 500 μm zone immediately surrounding the GA lesion showed the strongest correlation with GA enlargement rate [17, 18]. We have speculated that a difference in the CC FD processing approach, including specifically the slab position, may have contributed to these inconsistent results. As the optimal slab position with regards to clinical utility remains undefined, it may be useful to establish which slab position demonstrates the best correlation with GA enlargement.

In our study, we quantitatively assessed the CC/inner choroidal flow deficits using different axial slab positions and correlated the resultant CC FD% with the enlargement rate of GA lesions in eyes with AMD.

## Methods

This retrospective study included patients diagnosed with geographic atrophy (GA) secondary to age-related macular degeneration who presented to the Doheny Eye Institute between 2016 and 2018. The study was conducted in compliance with the Health Insurance Portability and Accountability Act and the principles of the Declaration of Helsinki. The institutional review board (IRB) of the University of California – Los Angeles (UCLA) approved the study and waived the requirement for informed consent from study subjects because of the retrospective nature of the study.

Optical coherence tomography (OCT) and optical coherence tomography angiography (OCTA) images obtained on the Cirrus HD-OCT device (Carl Zeiss Meditec, Dublin, CA, USA) were collected from patients with GA. To be included in this study, patients had to have a baseline 6 × 6 mm OCTA scan and two 6 × 6 mm OCT volume scans one at the baseline visit, and the second one after at least 12 months.

The diagnosis of GA was affirmed by two independent certified Doheny Reading Center graders using OCT criteria established by the classification of atrophy meetings group for complete RPE and outer retinal atrophy (cRORA) [19]. These cRORA criteria consist of a hypertransmission zone ≥250 µm with attenuation or disruption of the RPE band ≥250 µm, with associated degeneration of the overlying photoreceptors. Patients were excluded from the study if they have CNV, high myopia greater than 6 dioptres, other macular disorders, previous intravitreal injections or vitrectomy surgery, poor quality scans (signal strength index <7) and/or significant motion artifact.

The Cirrus spectral domain OCT system includes a laser source characterized by a central wavelength of 840 nm and bandwidth of 90 nm that generates 68,000 A-scans per second. A-scan depth is 2 mm in tissue with an axial resolution of 5 μm and a lateral resolution of approximately 15 μm. After pupillary dilation, 6 × 6 mm structural OCT volume cubes (512 A-scans × 128 B-scans) were acquired centred on the fovea. For OCTA, 6 × 6 mm scans were also acquired, and were composed of 350 A-scans × 350 B-scans. OCTA flow was detected by analysis of signal decorrelation between sequential B-scans acquired at the same location using the complex optical microangiography (OMAG) algorithm [20]. Motion artifacts were mitigated by the use of retinal tracking software during image acquisition. A semi-automated retinal layer segmentation algorithm was applied to segment various retinal layers, including the RPE band [13]. The segmentation boundaries were meticulously reviewed by experienced Doheny Reading Center graders (AA, IB) and manual adjustment of segmentation errors was completed in all required B-scans to ensure accurate segmentation. The en face OCTA images of CC were produced by applying a maximum projection on the segmented volumes. The projection artifact removal tool of the device was applied to CC flow images to remove the flow signal projected from the overlying retinal vessels, in order to mitigate the impact of projection artifacts on quantitative assessment of CC FD%. The final en face CC image was exported for further analysis (1024 × 1024 pixels).

### Slab selection

For this study, the en face OCTA CC images were exported from three different slab locations. The inner and outer boundaries of the CC slab were adjusted relative to the RPE-fit line to generate three different axial positions; 11–21 μm, 21–31 μm and 31–41 μm from the RPE band centreline.

### Image processing

Signal compensation strategy was performed to compensate for the attenuation of flow signal in locations underlying pathological abnormalities in the retinal pigment epithelium/Bruch’s membrane complex. In brief, an inverse transformation was applied to the en face structural image and a Gaussian smoothing filter (3 × 3 pixel kernel) was utilized to reduce the speckle noise. The en face OCTA CC image was then multiplied by the resultant, inverted, smoothed, corresponding structural image [21]. Image analysis was performed by ImageJ software version 1.50 (National Institutes of Health, Bethesda, Maryland, USA).

### Quantitative image analysis

**Fig. 1:**
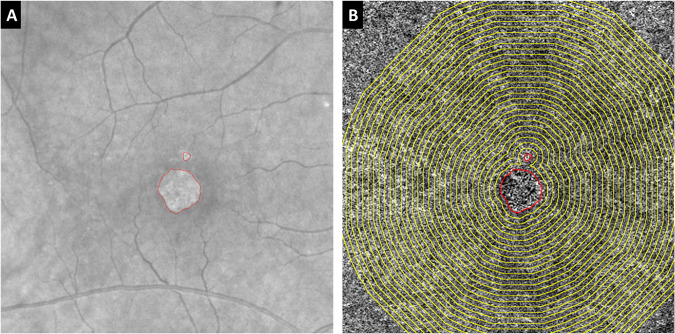
Creation of successive 100 μm wide concentric rings around the geographic atrophy lesion. Geographic atrophy (GA) margin was manually segmented on the en face structural fundus image of the entire volume (**A**) and successive 100 µm wide concentric rings were generated around the GA lesion using the ‘Edit – Selection – Enlarge’ function of ImageJ. The resultant rings were then applied to the corresponding OCT angiography en face 6 × 6 mm image of the choriocapillaris (**B**) in order to quantify the flow deficit percentage (FD%) in each ring.

The GA border was delineated on the baseline en face structural fundus image which represents reconstruction of all signals coming from the A-scans, and the corresponding B-scans [22]. Successive, 100 µm wide, concentric rings were generated around the GA lesion border using the ‘Edit – Selection – Enlarge’ tool in ImageJ software, which automatically creates a border that follows the boundary of the GA lesion. The generated grid of rings, which is distinctive to each case, was applied to the compensated CC images at the same position around the GA lesion (Fig. 1).

The 6 × 6 mm CC OCTA images (1024 × 1024 pixels) were binarized in order to calculate the FD% in each ring using the Phansalkar’s local thresholding strategy with a 3-pixel radius (approximately 17.6 microns) [23]. The images were then analysed with the “Analyze Particles” function. In accordance with previous reported methods, FDs with a diameter less than 24 μm were excluded from the analysis, as they are thought to fall within the normal range of spacing between choriocapillary vessels [24].

**Fig. 2:**
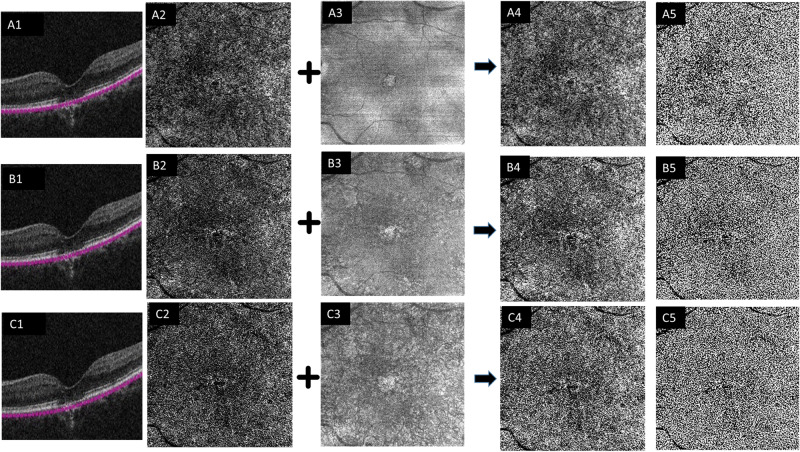
Structural B-scans (1) with segmentations at different levels. The inner and outer boundaries of the CC slab were adjusted relative to the RPE fit reference to generate three different axial positions; 11–21 μm (**A**), 21–31 μm (**B**) and 31–41 μm (**C**) from the RPE band centreline. OCT angiography (OCTA) en face 6 × 6 mm slabs of choriocapillaris (CC) (2) and corresponding structural en face slabs (3) exported from three different slab locations were used to generate a compensated CC image (4) that was the used to generate a binarized image (5) to calculate the CC flow deficit percentage.

The entire process was repeated for the OCTA CC images exported from the three different slab locations; 11–21, 21–31 and 31–41 µm from the RPE-fit line (Fig. 2).

The entire analysis was conducted on all image data by two masked, independent Doheny Reading Center graders (AA, IB) to assess the repeatability for all measurements.

### GA yearly enlargement rate calculation

The GA border was segmented on the en face OCT fundus image of baseline and follow-up scans to measure the GA lesion size at both visits. Square root transformation was utilized to exclude the confounding effect of baseline lesion size on the GA enlargement rates [16, 17]. Thus, using this method, yearly enlargement rate (yER; in units of mm) = $\frac{\sqrt{{GA}\,2}-\sqrt{{GA}\,1}}{{FU}}$. GA 2 and GA 1 represent GA area measurement at the follow-up and baseline visits, respectively (in units of square millimetres); and FU is the follow-up period (in years).

### Statistics

Statistical analyses were performed using SPSS Statistics version 20 (IBM, Armonk, NY). Test for normality of the data was performed using the Kolmogorov-Smimov and Shapiro-Wilk analyses. The spearman’s correlation coefficient was utilized to calculate the correlation between the FD% in each ring and the GA yER. Generalized estimating equations were utilized to adjust for the correlation between the two eyes of the same subject. Intraclass correlation coefficients (ICCs) were calculated to assess the agreement between the two grader’s measurements. A *P* value < 0.05 was considered to be statistically significant. With a sample size of 31 eyes, the study was 80% powered with a type I error of 0.05 and type II error of 0.20.

## Results

The study included Thirty-one eyes of 31 patients. Only 6 of the subjects were males. The mean age (SD) was 80.34 (6.28) years. The median follow-up time was 1.31 years (interquartile range, IQR: 1.22–1.37). The median subfoveal choroidal thickness was 187.51 µm (IQR: 128.72–234.25). The median baseline GA area was 0.69 mm^2^ (IQR: 0.25–3.15), and increased at the follow-up visit to a median of 1.35 mm^2^ (interquartile range: 0.44–5.41). After square root transformation, the median yearly enlargement rate of GA was 0.17 mm (IQR: 0.09–0.29).

The median FD% of the 11–21 µm slab was 41.88 (IQR: 35.47–44.02), which was significantly higher than the median FD% of the 21–31 µm slab; 29.20 (IQR: 26.65–35.19) (*p* = 0.001). Also, the median FD% of the 21–31 µm slab was significantly higher than the 31–41 µm slab; 26.61 (IQR: 24.25–28.54) (*p* = 0.002).

For the 11–21 µm slab, none of the rings (at any distances from the GA border) showed a significant correlation between the CC FD% and the yER (*p* > 0.05) (Supplementary Table 1).

For the 21–31 µm and 31–41 µm slab, the FD% in the first six rings (i.e. within the 600 µm region surrounding the GA) showed a statistically significant correlation with the yER (*p* < 0.05). However, rings beyond this 600 µm region did not show significant correlation between the FD% and the yearly enlargement rate (Supplementary Table 2, 3).

### Repeatability evaluation

The Interclass correlation coefficient of the yER measurement between the two graders was 0.984 (95% confidence interval (CI): 0.962–0.991) while the FD% measurement had an ICC of 0.905 (95% CI: 0.866–0.959).

## Discussion

In this study, we evaluated the effect of modulating the location of the en face OCTA CC slab on the correlation between choriocapillaris flow deficits and the yearly enlargement rate of GA lesions. We found the presence of this correlation was impacted by the axial position of the slab. In the most superficial slab that we evaluated (11–21 µm from the RPE-fit line), the FD% did not correlate with the rate of GA progression in any of the rings that we evaluated. However, in the other two deeper slabs (21–31 µm and 31–41 µm from the RPE-fit line), the FD% in the first six rings (within 600 µm from the GA margin) was significantly correlated with rate of geographic atrophy progression.

Assessment of the CC using OCTA has recently become a topic of great interest, and there are many papers that have evaluated the role of the CC in the context of several retinal and choroidal diseases [2, 4–7, 9, 17, 18].

However, one of the main limitations of current CC analysis approaches is the lack of a universally accepted ground-truth validation standard for the CC slab location. Although we have histologic information regarding the choriocapillaris, with vascular structures, there can be significant tissue processing artifact which could potentially impact the calculation of intercapillary spaces based on histology. Ideally, we would have adaptive optics -based high-resolution indocyanine green angiography to provide this missing ground truth information, but that is not yet available. In the absence of ground truth validation, the “optimal” analysis approach may be the one that yields a repeatable “CC” FD result, but still is sufficiently sensitive and specific enough to discriminate a clinical outcome of interest. For example, in the context of the present study, the CC FD processing approach which yields the best prediction of GA enlargement may be desirable – particularly if the observation can be replicated in other study cohorts.

Nassisi et al. studied CC flow deficits in two 500 μm wide rings surrounding GA, using a 31–41 μm slab, and reported greater CC impairment in the para-atrophy ring adjacent to GA border [9]. In addition, flow deficits in the para-atrophy ring, using the same slab location, were correlated with yearly GA enlargement rate [17, 18]. These findings would appear to be harmonious with histopathological studies in post-mortem GA eyes. Biesemeier et al. [25] used both light and electron microscopy to demonstrate the presence of CC impairment under an intact RPE layer and proposed that CC impairment might be the triggering event in GA development. Moreover, Li et al. [15] classified CC degeneration in GA eyes into five stages. In the first stage, CC fully occupies the area between intercapillary pillars of BM. In the second stage, CC occupies less than 50% of the area. In the third stage, the endothelium of the CC has disappeared and phagocytes occupy the spaces in between CC. In the fourth stage, phagocytes have disappeared. In the fifth stage, the intercapillary pillars of BM has also disappeared. The authors reported a gradual increase of these abnormal CC phenotypes as one progresses from the non-atrophic to the atrophic sides of GA border, and they hypothesized that the endothelium of CC is required for maintaining its own basal lamina and also the outer layer of BM.

Pfau et al. [26] studied impairment of function in GA eyes by measuring retinal sensitivity surrounding GA using mesopic and dark-adapted two-colour fundus-controlled perimetry. They reported a gradual reduction in retinal sensitivity towards the GA border, as well as rod and cone photoreceptor dysfunction in the junctional zone. Thus, there is compelling structural and functional data to highlight extensive derangement of the outer retina-RPE-CC complex bordering these GA lesions. Stetson and colleagues [27] demonstrated that regions of photoreceptor abnormality surrounding the GA lesions could predict subsequent enlargement of these GA lesions. Given the close relationship between the photoreceptors, RPE, and CC, it is perhaps not surprising that the CC surrounding the GA lesion also predicts the enlargement of these lesions.

The CC is a dense capillary network that is arranged as hexagonal-shaped lobules. The thickness of CC layer is about 10 μm in the fovea and 7 μm in the periphery. The thickness of RPE cells is approximately 14 μm in the central part of the retina and not uniform across the macula, and the BM is about 2–4 μm [28]. The RPE reference line of the Cirrus HD-OCT device is automatically segmented at the middle of RPE/BM complex. Based on these values, one might anticipate that the CC would be positioned approximately 10 microns below RPE centreline, and thus the 11–21 slab (i.e. offset 11 microns below the RPE band centre and extending 10 microns axially) would seem to be most anatomically correct.

However, Byon et al. [14] reported that a superficial slab which is close to this presumed correct anatomical position of CC is more susceptible to segmentation errors which leads to accidental inclusion of the BM-RPE complex within the segmented slab. In contrast, they observed that deeper slabs appeared to be less susceptible to these minimal segmentation errors. These findings are in agreement with our results, as we found a higher CC FD% in the 11–21 μm than the 21–31 μm slab and higher CC FD% in the 11–21 μm than the 31–41 μm slab. The loss of correlation between the FD% in the first six rings and GA progression in the superficial (11–21 μm) slab might be in part due to subtle segmentation errors which might lead to variable amounts of BM/RPE inclusion in the segmented CC slab, but were not detectable or apparent to the graders despite careful review. Therefore, one might hypothesize that deeper slabs are getting more accurate and reliable quantitative metrics as a result of the reduction of the effect of segmentation errors.

In contrast to our findings, Thulliez et al. [16] noted that global CC FD% was better correlated with GA enlargement than those in the region immediately surrounding GA. They used a superficially located slab which was 20 μm thick and the inner boundary was located just beneath BM. One wonders whether the results of their study, which included a smaller cohort than ours, may have also been impacted by subtle segmentation errors which may have been more difficult to detect in regions adjacent to the GA lesion compared to more remote areas.

Our study also has some limitations which should be taken into consideration. First, the study is retrospective and as a result may be susceptible to ascertainment bias. Second, the sample size is still relatively small. Our findings, will require validation in a larger and independent cohort. On the other hand, the main focus of the present study was to highlight that the processing approach with regards to slab selection can impact CC FD quantification and can impact the interpretation of study results. Third, the GA lesion border was established based on *en face* OCT using the recent CAM criteria. At present, fundus autofluorescence is the primary imaging modality for quantifying GA lesions in clinical trials. However, recent studies have highlighted that structural OCT may be a reliable imaging modality for detecting GA [29]. Moreover, Yehoshua et al. [30] reported that SD-OCT can reliably measure the area and enlargement rate of GA. The final limitation of our study is the utilization of spectral domain-OCTA device which has a short 840 nm wavelength and lack of enhanced depth imaging. This may result in attenuation of the CC signal under drusen and areas of pathological RPE alterations. However, we addressed this potential limitation by incorporating the signal compensation method [21].

In summary, we observed that the superficial CC slab immediately beneath the RPE/BM complex in the presumed anatomic location of the CC did not show a correlation between the severity of the CC FD and progression of GA. In contrast, deeper inner choroidal slabs were able to identify this correlation. These findings highlight the importance of slab selection in CC analyses on OCTA, and would suggest that multiple CC slabs may need to be performed until an optimal, consensus OCTA processing approach can be defined.

## Summary

### What was known before

- In this study, we evaluated the effect of modulating the location of the en face OCTA CC slab on the correlation between choriocapillaris flow deficits and the yearly enlargement rate of GA lesions. We found the presence of this correlation was impacted by the axial position of the slab. In the most superficial slab that we evaluated (11–21 µm from the RPE-fit line), the FD% did not correlate with the rate of GA progression in any of the rings that we evaluated. However, in the other two deeper slabs (21–31 µm and 31–41 µm from the RPE-fit line), the FD% in the first five rings (within 500 µm from the GA margin) was significantly correlated with rate of geographic atrophy progression.

### What this study adds

- We observed that the superficial CC slab immediately beneath the RPE/BM complex in the presumed anatomic location of the CC did not show a correlation between the severity of the CC FD and progression of GA. In contrast, deeper inner choroidal slabs were able to identify this correlation. These findings highlight the importance of slab selection in CC analyses on OCTA, and would suggest that multiple CC slabs may need to be performed until an optimal, consensus OCTA processing approach can be defined.

### Supplementary information

Supplementary Table 1

Supplementary Table 2

Supplementary Table 3

## Data Availability

The data that support the findings of this study are available on request from the corresponding author.
