# Supplementary material for: Impact of slab selection on the relationship between choriocapillaris flow deficits and enlargement rate of geographic atrophy
Source: Eye (Lond). 2023 Oct 21;38(5):847–52. doi: 10.1038/s41433-023-02788-2 (PMC10966059; doi:10.1038/s41433-023-02788-2)
Supplement: Supplementary file 1 — Supplementary Table 1 [file 41433_2023_2788_MOESM1_ESM.docx]

| Distance from GA margin (µm) | Flow deficit%  Median (interquartile range) | Correlation  coefficient | p value | |
| --- | --- | --- | --- | --- |
| 100 | 44.46 (40.70 – 52.52) | 0.15 | 0.38 | |
| 200 | 44.37 (41.36 – 53.44) | 0.20 | 0.22 | |
| 300 | 44.76 (40.21 – 50.65) | 0.13 | 0.43 | |
| 400 | 44.56 (39.82 – 49.38) | 0.16 | 0.33 | |
| 500 | 44.68 (39.71 – 49.89) | 0.16 | 0.35 | |
| 600 | 45.41 (40.88 – 48.17) | 0.12 | 0.49 | |
| 700 | 44.82 (39.18 – 47.74) | 0.14 | 0.40 | |
| 800 | 44.75 (38.47 – 46.60) | 0.04 | 0.82 | |
| 900 | 43.59 (38.20 – 46.64) | 0.08 | 0.64 | |
| 1000 | 43.17 (38.55 – 45.72) | 0.06 | 0.72 | |
| 1100 | 41.94 (38.19 – 45.69) | 0.06 | 0.74 | |
| 1200 | 41.40 (37.93 – 45.25) | 0.10 | 0.57 | |
| 1300 | 41.28 (37.95 – 44.19) | -0.02 | 0.90 | |
| 1400 | 40.94 (37.25 – 45.07) | 0.02 | 0.92 | |
| 1500 | 40.37 (37.45 – 44.79) | 0.01 | 1.00 | |
| 1600 | 40.19 (36.87 – 43.93) | -0.06 | 0.75 | |
| 1700 | 39.66 (35.11 – 43.85) | 0.03 | 0.86 | |
| 1800 | 38.75 (34.83 – 43.38) | -0.04 | 0.82 | |
| 1900 | 39.22 (34.04 – 43.48) | -0.01 | 0.97 | |
| 2000 | 37.68 (33.75 – 42.04) | 0.06 | 0.73 | |
| 2100 | 38.44 (33.46 – 41.52) | 0.02 | 0.90 | |
| 2200 | 37.59 (33.01 – 42.47) | 0.01 | 0.99 | |
| 2300 | 36.86 (32.45 – 40.93) | -0.04 | 0.81 | |
| 2400 | 36.19 (32.21 – 40.92) | 0.02 | 0.90 | |
| 2500 | 36.47 (31.82 – 40.39) | 0.01 | 0.97 | |
| 2600 | 36.95 (31.81 – 40.75) | -0.04 | 0.82 | |
| 2700 | 36.36 (31.62 – 40.95) | 0.08 | 0.67 | |
| 2800 | 36.26 (31.48 – 40.73) | 0.06 | 0.76 | |
| 2900 | 35.82 (31.65 – 42.20) | 0.02 | 0.93 | |
| 3000 | 36.86 (31.48 – 43.50) | 0.10 | 0.61 | |
| GA: Geographic atrophy. The distance value denotes the distance of the outer border of the 100 µm wide ring from the GA lesion border. | | | |  |

**Table 1: Correlation between the choriocapillaris flow deficit percentage of the 11 – 21 µm slab with the yearly enlargement rate of geographic atrophy lesions.**
