# Supplementary material for: Impact of slab selection on the relationship between choriocapillaris flow deficits and enlargement rate of geographic atrophy
Source: Eye (Lond). 2023 Oct 21;38(5):847–52. doi: 10.1038/s41433-023-02788-2 (PMC10966059; doi:10.1038/s41433-023-02788-2)
Supplement: Supplementary file 2 — Supplementary Table 2 [file 41433_2023_2788_MOESM2_ESM.docx]

| Distance from GA margin (µm) | Flow deficit%  Median (interquartile range) | Correlation  coefficient | p value | |
| --- | --- | --- | --- | --- |
| 100 | 38.54 (31.05 – 43.55) | 0.481 | 0.01 | |
| 200 | 37.69 (32.28 – 43.78) | 0.470 | 0.01 | |
| 300 | 36.84 (32.62 – 43.83) | 0.421 | 0.01 | |
| 400 | 37.27 (29.64 – 43.21) | 0.383 | 0.02 | |
| 500 | 35.77 (30.36 – 41.87) | 0.382 | 0.02 | |
| 600 | 34.05 (29.48 – 40.01) | 0.348 | 0.03 | |
| 700 | 34.79 (27.33 – 39.75) | 0.313 | 0.06 | |
| 800 | 34.35 (26.41 – 38.15) | 0.24 | 0.14 | |
| 900 | 33.65 (27.52 – 36.22) | 0.28 | 0.09 | |
| 1000 | 32.20 (26.43 – 36.86) | 0.23 | 0.17 | |
| 1100 | 30.82 (26.58 – 35.83) | 0.21 | 0.22 | |
| 1200 | 31.04 (27.01 – 35.07) | 0.19 | 0.26 | |
| 1300 | 30.08 (26.87 – 34.42) | 0.13 | 0.44 | |
| 1400 | 28.92 (26.43 – 34.52) | 0.12 | 0.48 | |
| 1500 | 28.84 (26.14 – 33.18) | 0.09 | 0.59 | |
| 1600 | 28.81 (26.01 – 31.82) | 0.03 | 0.86 | |
| 1700 | 27.94 (24.64 – 30.99) | 0.02 | 0.92 | |
| 1800 | 27.87 (24.08 – 31.15) | -0.02 | 0.89 | |
| 1900 | 27.02 (23.82 – 31.75) | 0.01 | 0.95 | |
| 2000 | 26.04 (24.09 – 29.46) | 0.05 | 0.77 | |
| 2100 | 25.88 (23.82 – 30.42) | 0.09 | 0.60 | |
| 2200 | 24.94 (21.69 – 30.26) | 0.05 | 0.77 | |
| 2300 | 24.89 (23.04 – 27.97) | -0.02 | 0.92 | |
| 2400 | 24.33 (22.61 – 29.28) | 0.09 | 0.64 | |
| 2500 | 24.65 (21.67 – 28.52) | 0.09 | 0.64 | |
| 2600 | 24.33 (21.90 – 28.08) | 0.03 | 0.89 | |
| 2700 | 24.04 (22.33 – 29.87) | 0.11 | 0.54 | |
| 2800 | 24.05 (22.36 – 31.55) | 0.03 | 0.89 | |
| 2900 | 24.44 (21.40 – 28.54) | 0.01 | 0.99 | |
| 3000 | 24.57 (21.50 – 28.83) | 0.10 | 0.61 | |
| GA: Geographic atrophy. The distance value denotes the distance of the outer border of the 100 µm wide ring from the GA lesion border. | | | |  |

**Table 2: Correlation between the choriocapillaris flow deficit percentage of the 21 – 31 µm slab with the yearly enlargement rate of geographic atrophy lesions.**
