# Supplementary material for: Impact of slab selection on the relationship between choriocapillaris flow deficits and enlargement rate of geographic atrophy
Source: Eye (Lond). 2023 Oct 21;38(5):847–52. doi: 10.1038/s41433-023-02788-2 (PMC10966059; doi:10.1038/s41433-023-02788-2)
Supplement: Supplementary file 3 — Supplementary Table 3 [file 41433_2023_2788_MOESM3_ESM.docx]

| Distance from GA margin (µm) | Flow deficit%  Median (interquartile range) | Correlation  coefficient | p value | |
| --- | --- | --- | --- | --- |
| 100 | 33.10 (30.40 – 36.60) | 0.55 | 0.001 | |
| 200 | 32.38 (28.60 – 35.39) | 0.52 | 0.001 | |
| 300 | 31.06 (27.27 – 32.61) | 0.42 | 0.009 | |
| 400 | 31.12 (28.22 – 34.83) | 0.40 | 0.012 | |
| 500 | 29.79 (26.48 – 34.04) | 0.35 | 0.029 | |
| 600 | 27.95 (25.29 – 32.01) | 0.37 | 0.021 | |
| 700 | 27.88 (24.33 – 31.93) | 0.32 | 0.051 | |
| 800 | 27.51 (23.47 – 30.97) | 0.23 | 0.16 | |
| 900 | 26.17 (23.12 – 29.50) | 0.17 | 0.31 | |
| 1000 | 27.11 (23.71 – 29.51) | 0.16 | 0.34 | |
| 1100 | 26.83 (24.05 – 29.09) | 0.13 | 0.45 | |
| 1200 | 26.45 (24.15 – 28.50) | -0.02 | 0.99 | |
| 1300 | 26.43 (24.33 – 29.15) | -0.04 | 0.80 | |
| 1400 | 26.47 (24.04 – 28.94) | 0.06 | 0.75 | |
| 1500 | 26.06 (23.76 – 28.14) | -0.10 | 0.56 | |
| 1600 | 25.29 (23.20 – 28.28) | -0.20 | 0.24 | |
| 1700 | 25.20 (23.43 – 27.63) | -0.05 | 0.78 | |
| 1800 | 24.88 (22.23 – 28.01) | -0.09 | 0.59 | |
| 1900 | 24.58 (22.01 – 27.51) | -0.06 | 0.71 | |
| 2000 | 25.31 (20.98 – 27.64) | -0.08 | 0.65 | |
| 2100 | 23.96 (21.53 – 26.99) | -0.02 | 0.92 | |
| 2200 | 24.37 (22.52 – 26.80) | -0.05 | 0.80 | |
| 2300 | 24.43 (22.25 – 27.09) | -0.17 | 0.35 | |
| 2400 | 23.78 (22.09 – 27.32) | -0.15 | 0.34 | |
| 2500 | 25.01 (22.91 – 26.83) | -0.10 | 0.58 | |
| 2600 | 24.31 (21.98 – 26.41) | -0.11 | 0.57 | |
| 2700 | 25.05 (21.53 – 26.96) | -0.14 | 0.59 | |
| 2800 | 24.89 (22.29 – 27.28) | -0.13 | 0.49 | |
| 2900 | 24.89 (21.51 – 26.79) | -0.09 | 0.63 | |
| 3000 | 24.65 (20.62 – 26.74) | -0.05 | 0.79 | |
| GA: Geographic atrophy. The distance value denotes the distance of the outer border of the 100 µm wide ring from the GA lesion border. | | | |  |

**Table 3: Correlation between the choriocapillaris flow deficit percentage of the 31 – 41 µm slab with the yearly enlargement rate of geographic atrophy lesions.**
